# SARS-CoV-2-Specific Antibody (Ab) Levels and the Kinetic of Ab Decline Determine Ab Persistence Over 1 Year

**DOI:** 10.3389/fmed.2022.822316

**Published:** 2022-02-15

**Authors:** Erika Garner-Spitzer, Angelika Wagner, Michael Kundi, Hannes Stockinger, Anna Ohradanova-Repic, Laura Gebetsberger, Anna-Margarita Schoetta, Venugopal Gudipati, Johannes B. Huppa, Renate Kunert, Patrick Mayrhofer, Thomas R. Kreil, Maria R. Farcet, Eva Hoeltl, Ursula Wiedermann

**Affiliations:** ^1^Institute of Specific Prophylaxis and Tropical Medicine, Center for Pathophysiology, Infectiology and Immunology, Medical University of Vienna, Vienna, Austria; ^2^Center for Public Health, Medical University of Vienna, Vienna, Austria; ^3^Institute for Hygiene and Applied Immunology, Center for Pathophysiology, Infectiology and Immunology, Medical University of Vienna, Vienna, Austria; ^4^Department of Biotechnology, University of Natural Resources and Life Sciences, Vienna, Austria; ^5^Global Pathogen Safety, Baxter AG, a Takeda company, Vienna, Austria; ^6^Health Center Erste Bank, Erste Bank, Vienna, Austria

**Keywords:** SARS-CoV-2, infection, neutralizing Abs, antibody kinetics, vaccination, persistence

## Abstract

In a SARS-CoV-2 seroprevalence study conducted with 1,655 working adults in spring of 2020, 12 of the subjects presented with positive neutralization test (NT) titers (>1:10). They were here followed up for 1 year to assess their Ab persistence. We report that 7/12 individuals (58%) had NT_50 titers ≥1:50 and S1-specific IgG ≥50 BAU/ml 1 year after mild COVID-19 infection. S1-specific IgG were retained until a year when these levels were at least >60 BAU/ml at 3 months post-infection. For both the initial fast and subsequent slow decline phase of Abs, we observed a significant correlation between NT_50 titers and S1-specific IgG and thus propose S1-IgG of 60 BAU/ml 3 months post-infection as a potential threshold to predict neutralizing Ab persistence for 1 year. NT_50 titers and S1-specific IgG also correlated with circulating S1-specific memory B-cells. SARS-CoV-2-specific Ab levels after primary mRNA vaccination in healthy controls were higher (Geometric Mean Concentration [GMC] 3158 BAU/ml [CI 2592 to 3848]) than after mild COVID-19 infection (GMC 82 BAU/ml [CI 48 to 139]), but showed a stronger fold-decline within 5–6 months (0.20–fold, to GMC 619 BAU/ml [CI 479 to 801] vs. 0.56–fold, to GMC 46 BAU/ml [CI 26 to 82]). Of particular interest, the decline of both infection- and vaccine-induced Abs correlated with body mass index. Our data contribute to describe decline and persistence of SARS-CoV-2-specific Abs after infection and vaccination, yet the relevance of the maintained Ab levels for protection against infection and/or disease depends on the so far undefined correlate of protection.

## Introduction

We recently reported in a longitudinal seroprevalence study conducted in a cohort of working adults (*n* = 1,655) that SARS-CoV-2 RBD-specific antibodies (Abs) persist for at least 6 months independent of symptom severity; we further observed that COVID-19 symptoms anosmia and/or dysgeusia correlated most closely with the detection of virus-neutralizing Abs (1). Also others have shown that COVID-19 symptom severity and in particular loss of taste and/or smell were associated with induction of higher Ab levels which persisted for up to 8 months after infection (2–4). In order to assess SARS-CoV-2 seroprevalence in the investigated cohort at the end of the third pandemic wave in Austria and to determine Ab persistence over 1 year, the study subjects were invited to a follow up blood draw between mid-April and mid May 2021. Of the 1,655 subjects recruited in April 2020 only 12 presented with neutralization test (NT) titers >1:10 and were now followed up for the kinetic and long-term persistence of Abs and memory B cells for 1 year.

Quantitative evaluation of SARS-CoV-2-specific Abs with virus neutralization test (NT_50 1: x), RBD-specific IgG ELISA (IU/ml) and S1-specific IgG ELISA (BAU/ml) and semi-quantitative testing with surrogate-virus neutralization test (sVNT) (% inhibition) and NCP-specific IgG ELISA (ratios) were performed in serum samples obtained 1, 3, 6 months and 1 year after their mild COVID-19 infection. Cellular responses after 1 year were assessed by quantification of circulating S1-specific B memory cells. In parallel, healthy controls enrolled in a SARS-CoV-2 vaccination study at our institute received 2 doses of an mRNA vaccine and the obtained Ab results up to 6 months after the 2nd vaccine dose allowed the comparison of S1-specific IgG Ab levels and decline kinetics in infected vs. vaccinated subjects (*n* = 42).

We here show that the initial levels of SARS-CoV-2-specific Abs after infection and the kinetics of their decline determined the persistence over 1 year. While vaccinated individuals showed significantly higher Ab levels than infected, the fold-decline of Abs was accordingly stronger after vaccination than infection, but correlated in both groups with body mass index.

## Materials and Methods

### Study Participants

The participants of the seroprevalence study were employees from a large Viennese company and obtaining written informed consent, blood draws and completion of questionnaires was carried out at the on-site medical center. Whole blood samples were then delivered to the Institute of Specific Prophylaxis and Tropical Medicine where serum and PBMC samples were prepared. Healthy control subjects enrolled in an ongoing SARS-CoV-2 vaccination study received 2 doses of an mRNA vaccine (BNT1622b2 [BioNTech/Pfizer] or mRNA-1273 [Moderna]) at a 4 weeks interval. Blood for Ab measurements was taken before the first dose, on the day of the second dose and 4 weeks and 5–6 months after the 2nd dose. The studies were approved by the Ethics committee of the Medical University of Vienna (EK 1438/2020, EK 1746/2020, and EK 1073/2021).

### Preparation and Storage of Serum and PBMC

Serum samples were obtained from native venous blood and stored at −20°C until evaluation. Peripheral blood mononuclear cells (PBMC) were prepared from heparinized blood by Ficoll Paque density gradient centrifugation, as previously described (5) and immediately used for flow-cytometric assays and re-stimulation.

### SARS-CoV-2-Specific Ab Measurements

*SARS-CoV-2 neutralization (NT) assay* was performed as previously described (6). In brief, Vero cells (ATCC CCL-81) sourced from the European Collection of Authenticated Cell Cultures (84113001) were cultured in TC-Vero medium supplemented with 5% fetal calf serum, L-glutamine (2 mM), non-essential amino acids (1x), sodium pyruvate (1 mM), gentamicin sulfate (100 mg/ml), and sodium bicarbonate (7.5%). SARS-CoV-2 strain BetaCoV/Germany/Bav-Pat1/2020 was kindly provided by the Institute of Virology at Charité Universitätsmedizin, Berlin, Germany. For the SARS-CoV-2 neutralization assays, samples were serially diluted 1:2 and incubated with 100 tissue culture infectious dose 50% (TCID50) of SARS-CoV-2 per well. The samples were subsequently applied onto Vero cells seeded in tissue culture plates and incubated for 5 to 7 days, after which the cells were evaluated for the presence of a cytopathic effect and the SARS-CoV-2 neutralization titer (NT_50), i. e. the reciprocal sample dilution resulting in 50% virus neutralization, was determined using the Spearman-Kärber formula and reported as 1:x. Cut-off values varied between 1:3 and 1:7.7 NT_50 between the assay runs, depending on sample-predilution and level of sample cytotoxicity.

*SARS-CoV-2 RBD-specific IgG Abs* were determined with commercial ELISA (Beijing Wantai Biological Pharmacy Enterprise Co., Ltd., Beijing, China) according to manufacturer's instructions. Results were calculated as International Units (IU)/ml according to WHO International Standard (NIBSC code: 20/136).

*Anti-SARS-CoV-2-QuantiVac-ELISA (IgG)* obtained from Euroimmune®, Euroimmun Medizinische Labordiagnostik AG (Lübeck, Germany) was carried out according to the manufacturer's instructions to determine Spike 1-specific IgG concentrations and results were calculated as binding antibody units (BAU)/ml according to WHO International Standard (NIBSC code: 20/136).

*SARS-CoV-2-NeutraLISA surrogate virus neutralization test (sVNT)* obtained from Euroimmune®, Euroimmun Medizinische Labordiagnostik AG (Lübeck, Germany) was performed according to manufacturer's instructions and results were calculated as % inhibition with following interpretation: % inhibition <20—negative; % inhibition ≥20 to <35—borderline; % inhibition ≥35—positive). In brief, wells coated with SARS-CoV-2 S1/RBD domain were incubated with soluble ACE2 protein and diluted sera. Neutralizing Abs present in sera inhibited the binding of ACE2 to the S1/RBD proteins on the well surface and were quantified as % inhibition.

*SARS-CoV-2 NCP-specific IgG* (nucleocapsid protein) were quantified by ELISA (Euroimmune®, Euroimmun Medizinische Labordiagnostik AG, (Lübeck, Germany) carried out according to manufacturer's instructions; results were expressed as ratios (ratio < 0.8 negative, 0.8 to 1.1 borderline, >1.1 positive).

### Quantification of S1-Specific B Memory Cells

For detection of SARS-CoV-2 S1-specific B memory cells, biotinylated S1 protein antigen was individually mixed with streptavidin-APC and streptavidin-BV421 probes. Streptavidin-APC-Cy7 was used as a decoy probe to gate out SARS-CoV-2 non-specific streptavidin-binding B cells. The S1 antigen probes and decoy probe were mixed in Brilliant Buffer (BD Bioscience, Cat# 566349) containing 5 μM free D-biotin and subsequently 3 × 10^6^ PBMC prepared in U-bottom 96-well plates were stained with 50 μL antigen probe cocktail, containing 100 ng S1 per probe, at 4°C for 1 h. Then surface staining was performed with directly-labeled monoclonal Abs toward human CD19 (FITC, clone HIB19), human CD27 (PE, clone L128), human CD38 (PerCP-Cy5.5, clone HIT2) and human immunoglobulin D (IgD) (PE-Cy7, clone IA6-2), all BD Bioscience, in Brilliant Buffer at 4°C for 30 min. Dead cells were excluded by using Fixable viability dye eFluor-506 (eBioscience, now Thermo Fisher Scientific). Data were acquired on a FACS Canto II flow cytometer by gating on cells with forward/side light scatter properties of lymphocytes and analyzed with FACS Diva 8.0 software.

### Statistical Evaluation

Persistence of immunity after infection or vaccination is sometimes life long and it has been shown that decline kinetics of vaccination- or infection-induced Abs are best described by two-component models that are log-linear (7–10). The first component starts after the maximum Ab level is reached (a few weeks after the first booster vaccination or after symptoms have resolved in infected individuals) and leads to an initial fast decline. The second component typically starts after a few months and is often very slowly declining. We have applied such a model for the different antibody measurements.


(1) $$\begin{array}{r} \log y_{t}\;=\;\log y_{o}-\beta_{1}.t-{\beta_{2}.\left(t-\delta \right)}^{+} \end{array}$$

The equation (1) describes the antibody level y_t_ at time (*t*) months after the baseline time point (o) as a log linear function of t with a cut point δ (set at 6 months) when the slow component starts to operate (the indicator function x^+^ takes on zero of x <0 and is = x if x>0 for any real value x). Parameters ß_1_ and ß_2_ were estimated by a General Estimation Equation (GEE) model with an unstructured correlation matrix (i.e., allowing the estimation algorithm to account for serial correlation). In addition, the parameters were estimated for each participant separately.

## Results

For assessing SARS-CoV-2 seroprevalence at the end of the third pandemic wave in April 2021, 941 subjects could be followed up, but due to the start of the vaccination programs, a number of participants (*n* = 123) had to be excluded because of prior vaccination. In the evaluable cohort (*n* = 818) seroprevalence increased from app 0.1% in April 2020 to 12.84% in spring of 2021 (*n* = 105 of 818 subjects were seropositive).

**Figure 1:**
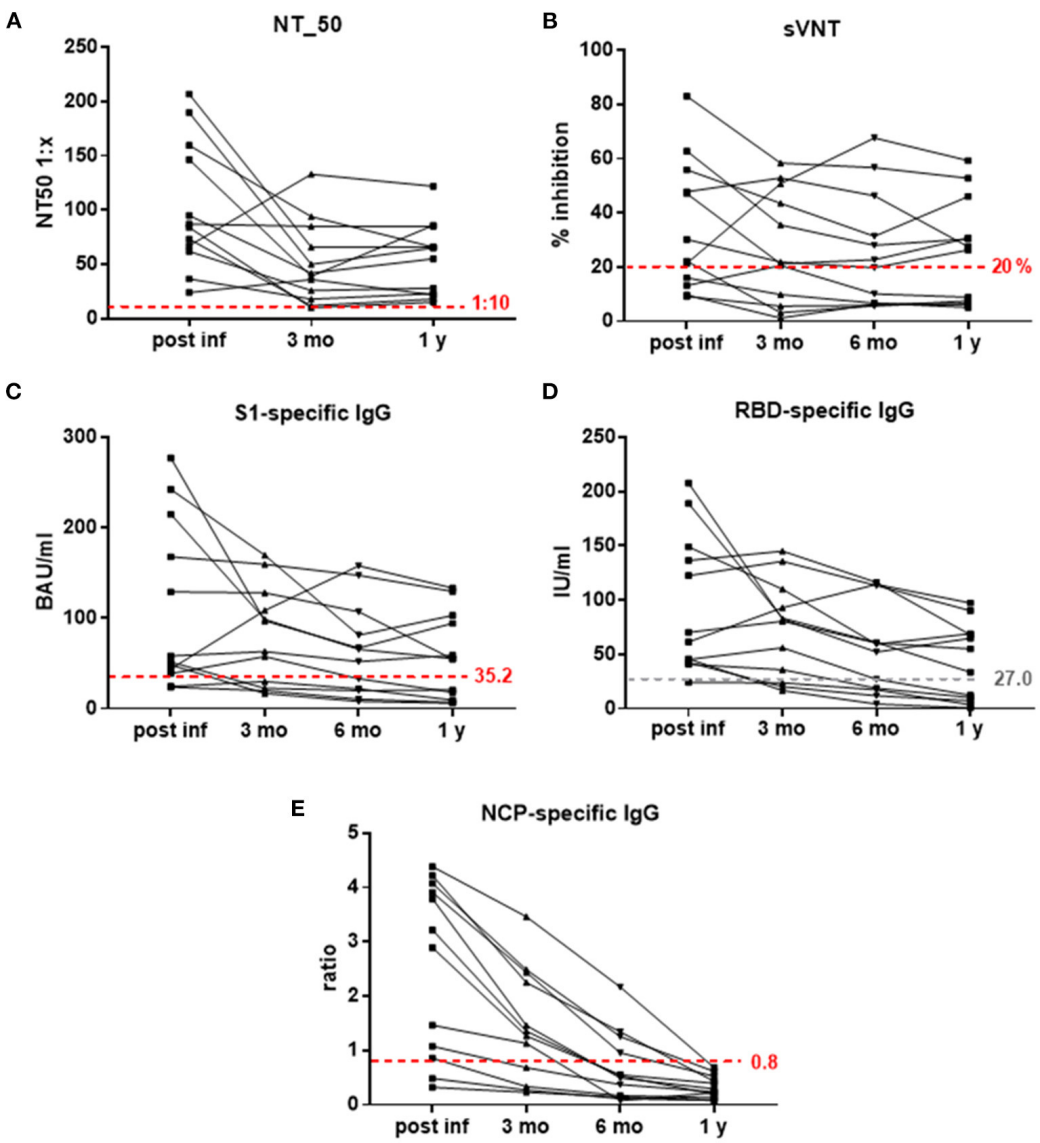
**(A–E)** Kinetics of SARS-CoV-2-specific Abs measured with different assays. **(A)** NT_50 titers 1:x, **(B)** % inhibition in sVNT (negative cut-off 20%), **(C)** S1-specific IgG in BAU/ml (positive cut-off 35.2), **(D)** RBD-specific IgG in IU/ml, and **(E)** NCP-specific IgG (ratios, negative cut-off 0.8) post-infection and 3 months, 6 months and 1 year thereafter; dashed red lines indicate positive (NT_50, S1-specific IgG) and negative (sVNT, NCP-specific IgG) cut-off values; dashed gray line—proposed positive cut-off for RBD-specific IgG. BAU, binding antibody units; IU, international antibody units; NT_50, reciprocal sample dilution resulting in 50% virus neutralization; mo, months; NCP, SARS-CoV-2 nucleo capside protein; RBD, SARS-CoV-2 receptor binding domain; S1, SARS-CoV-2 Spike protein 1; sVNT, surrogate-virus neutralization test; y, year.

For evaluation of Ab persistence, 12 participants who were infected in April 2020 were now followed up to 1 year. Measurement of SARS-CoV-2-specicfic Abs showed that 7 of 12 subjects (58%) had neutralizing Ab titers (NT_50) ≥ 1:50 1 year after their mild COVID-19 infection, but importantly, all 12 subjects retained NT_50 titers above the positive cut off 1:10. In accordance, S1-specific IgG concentrations ≥50 BAU/ml were detectable in these seven subjects, as were RBD-specific IgG Abs ≥34 IU/ml. Also the surrogate virus neutralization test showed a binding inhibition ≥26% in these individuals after 1 year (Figures 1A–D). In contrast, NCP-specific IgG Abs had declined below the negative cut-off in all investigated subjects after 1 year (Figure 1E).

**Figure 2:**
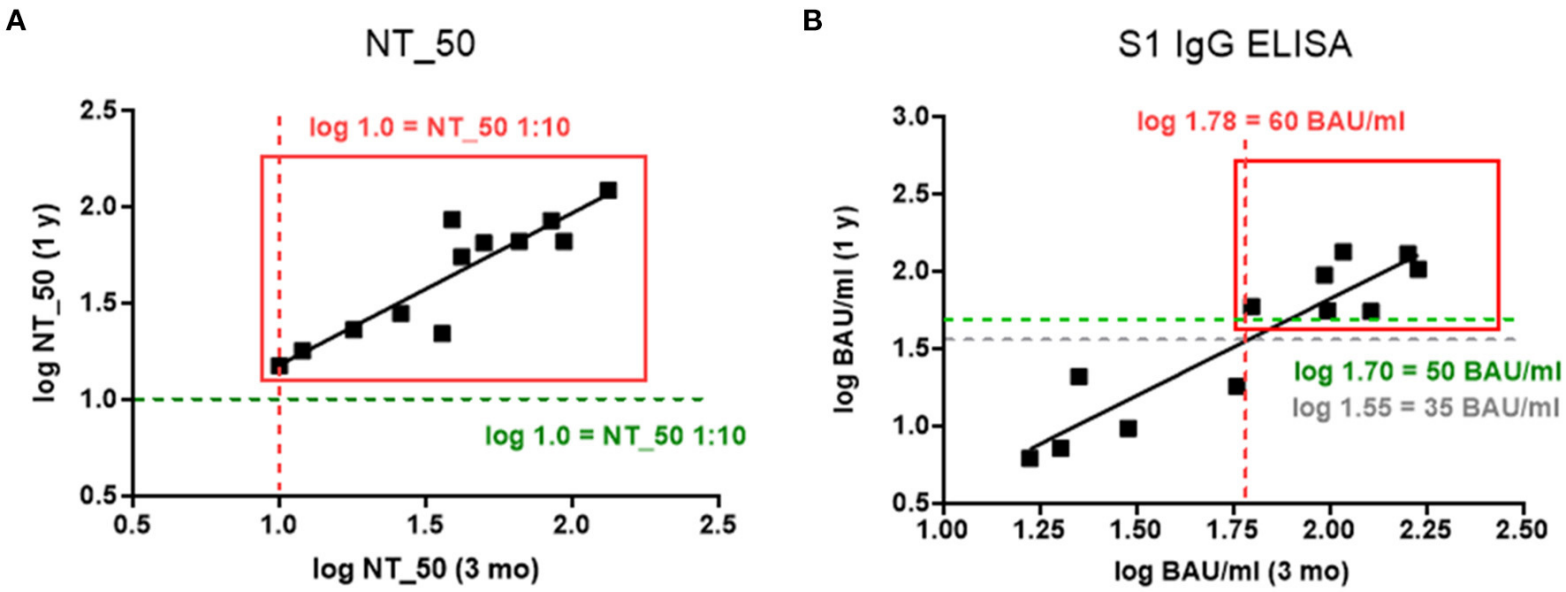
**(A,B)** Relationship between log transformed Ab concentrations measured 3 months and 1 year post infection for **(A)** NT_50 titers 1:x (Spearman correlation coefficient, and p-value for the test against r_s_ = 0: *r*_*s*_ = 0.861, *p* = 0.0006), and **(B)** S1-specific IgG in BAU/ml (*r*_*s*_ = 0.846, *p* = 0.0009). Gray lines indicate negative cut-off, red lines indicate potential thresholds for persistence at 3 months, and green lines indicate retained Ab concentrations after 1 year.

In order to define a threshold for the persistence of SARS-CoV-2-specific Abs, we correlated the Ab concentrations that were present at 3 months and 1 year post-infection. This showed that NT_50 titers above 1:10 at 3 months after infection remained stable for 1 year (*r*_s_= 0.861, *p* = 0.0006; Figure 2A). Regarding S1-specific IgG Abs, levels above 60 BAU/ml also declined only slightly to 50 BAU/ml after 1 year (*r*_s_= 0.846, *p* = 0.0009; Figure 2B).

**Figure 3:**
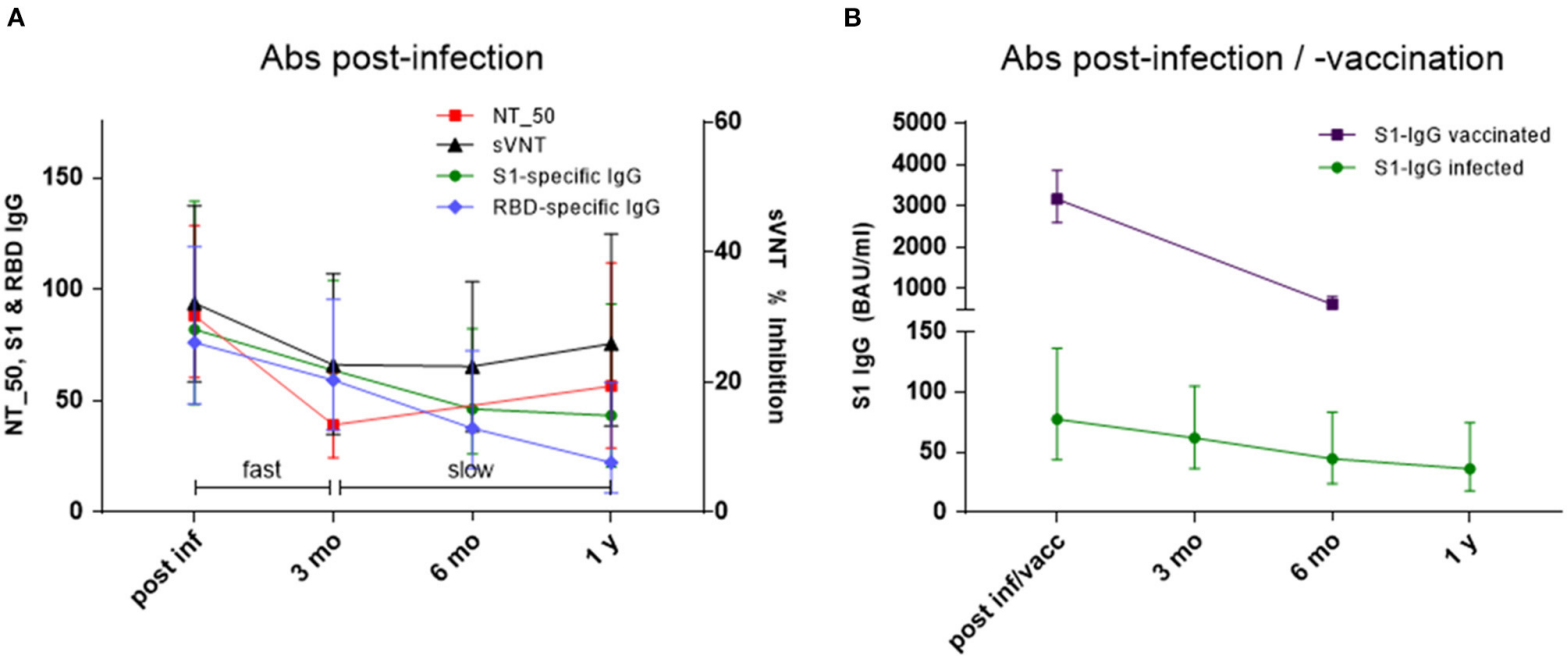
**(A,B)** Decline kinetics for **(A)** NT_50 titers 1: x, % inhibition in sVNT, S1-specific IgG in BAU/ml and RBD-specific IgG in IU/ml post-infection and 3 months, 6 months and 1 year thereafter. Geometric mean titers & 95% CI of NT_50 titers, geometric means & 95% CI of S1-specific IgG, RBD-specific IgG, and % inhibition in sVNT; fast corresponds to ß_1_ and slow to ß_2_ in Eq. 1 (see text); and **(B)** S1-specific IgG in BAU/ml (geometric mean & 95% CI) in infected individuals and healthy controls vaccinated twice (4-week interval) with SARS-CoV-2 mRNA vaccine (post-infection/vaccination and after 6 months).

To analyze the decline kinetics of the infection-induced Abs a two-component model was applied to the Ab levels measured with the respective assays. The decline kinetics for NT_50 titers, sVNT, S1-specific IgG and RBD-specific IgG are depicted in Figure 3A and indicated a fast decline phase up to 3 months after infection, followed by a slow decline phase from 3 months to 1 year. NT_50 titers correlated significantly with S1-specific IgG levels in both phases (fast: r_*s*_ = 0.596, *p* = 0.031; slow: r_*s*_ = 0.636, *p* = 0.019). With RBD-specific IgG correlation was seen only for the fast component (r_*s*_ = 0.602, *p* = 0.029). Interestingly, the two neutralizing assays NT_50 and sVNT had the most robust correlation for fast and slow component (fast: r_*s*_ = 0.648, *p* = 0.016; slow: r_*s*_ = 0.714, *p* = 0.006). This two-phase kinetic for Ab decline did not apply to NCP-specific IgG, which showed a rather continuing fast decline with GMC ratio falling below the negative threshold already within 6 months (Supplementary Figure 1).

Magnitude and decline kinetic of infection-induced S1-specific IgG were compared to those of vaccinated study participants (*n* = 42). In contrast to infected individuals, S1-specific IgG Abs reached very high levels after 2 doses of mRNA vaccine (GMC 3158 BAU/ml [CI 2592 to 3848] vs. GMC 82 BAU/ml [CI 48 to 139]), but the fold-decline over a period of 6 months was significantly faster in vaccinated (0.20-fold, to GMC 619 BAU/ml [CI 479 to 801]) vs. infected subjects (0.56-fold to GMC 46 BAU/ml [CI 26 to 82]) (Figure 3B). In two thirds of the vaccinated subjects (*n* = 26), also neutralization test titers were determined by live-virus assay. The NT_50 titers measured in serum samples obtained 4 weeks after the 2nd vaccination showed very good correlation with the respective S1-specific IgG results (*r*_*s*_ = 0.358, *p* = 0.0012). An interesting observation was that Ab decline rates correlated significantly with BMI in vaccinated individuals, and a similar trend was present in infected individuals (Supplementary Figures 2A,B).

Investigation of persistence of SARS-CoV-2 cellular immunity was done in selected subjects (*n* = 4) and we observed that the numbers of circulating S1-specific memory B cells related to NT_50 titers and levels of S1-specific IgG Abs (Table 1).

**Table 1:** S1-specific IgG in BAU/ml, NT_50 titers and respective percentages of B-cells, B memory cells and S1-specific B memory cells quantified by flow-cytometry for four COVID-19 convalescent subjects and one SARS-CoV-2 naïve control; y, year; ly, lymphocytes.

**Subject Nr**.	**Age (years)**	**Sex**	**BAU/ml (1y)**	**NT_50 1:x (1y)**	**B cells (% of ly)**	**B-memory cells (% of B)**	**% S1-specific B memory**
5	45–50	M	54.9	66	7.1	25.2	0.30
8	50–55	M	59.1	66	6.7	18.5	0.41
10	30–35	M	103.0	85	11.8	14.1	0.79
12	50–55	F	55.4	55	8.1	12.9	0.36
naive	40–45	M	–	–	4.2	25.9	0.01

## Discussion

Most reports on persistence of SARS-CoV-2-specific immune parameters show that NT titers and S1-specific IgG levels remain rather stable between 6 and 12 months after infection while NCP-specific Abs decline quickly (11–15). However, SARS-CoV-2-specific Ab persistence has so far not been investigated with regard to the decline kinetics of NT_50 titers and S1-specific IgG nor has a potential threshold been considered. This study showed that in order to determine for how long SARS-CoV-2-specific Abs are actually maintained, measurement of Abs 3 to 6 months after infection was crucial to assess persistence, which was determined by the maximal initial Ab levels and their decline kinetics. Our data indicate that fast and slow decline component of NT_50 titers and S1-specific IgG correlated; we thus propose that S1-specific IgG concentrations of 60 BAU/ml 3 months post-infection could be a potential threshold to predict maintenance of neutralizing Abs for 1 year. S1-specific IgG determined by ELISA are a technically more accessible parameter than NT assays and might therefore be better suited for routine measurement (16).

Data on persistence of vaccine-induced SARS-CoV-2 immunity are scarce, a recent report however gives a 5.49 adjusted odds ratio for hospitalization with COVID 19-like illness for infection- vs. mRNA vaccine-induced immunity after 90–179 days (17). Our results indicate that SARS-CoV-2-vaccine-induced Abs after 2-dose immunization were of much higher magnitude than those following mild infection (probably also depending on the type of vaccine), and showed a very rapid decline within 6 months. According to established decline kinetics, these Abs should be maintained at rather stable levels during the slow decline phase, but this has to be confirmed in future studies of long-term Ab persistence after SARS-CoV-2 vaccination. It was of particular interest that infection- and vaccine-induced Abs declined faster in overweight individuals, which is in accordance with reports for other vaccines (18, 19). Having in mind that obesity is one of the risk factors for a severe course of COVID-19 infection (20), the vaccination schedules for this risk group might be reconsidered.

The levels of maintained Abs in our recovered subjects related to numbers of persisting S1-specific B memory cells. It was shown that magnitude of infection-induced memory B cells correlates with subsequent vaccine-induced Ab levels (21) which indicates that important immunologic booster responses originate from these previously established memory B cells. This potentially leads to less stringent vaccination schedules in convalescent individuals.

Taken together, our data show that a threshold of SARS-CoV-2-specific Abs can be identified 3 months after (even) mild COVID-19 infection predicting persistence of neutralizing Ab levels for 1 year. We further report that SARS-CoV-2-specifc Abs after completed mRNA vaccination in healthy vaccinees were of higher magnitude than those after infection, but declined more rapidly. An observation of clinical importance is that both, infection- and vaccine-induced Abs showed faster decline in overweight individuals. It is important to stress, though, that the relevance of the maintained Abs for protection, also against variants of concern (VOC), can only be assessed once the correlate of protection has been defined.

## Data Availability Statement

The raw data supporting the conclusions of this article will be made available by the authors, without undue reservation.

## Ethics Statement

The studies involving human participants were reviewed and approved by Ethics Committee of the Medical University of Vienna (EK 1438/2020, EK 1746/2020, and EK 1073/2021). The patients/participants provided their written informed consent to participate in this study.

## Author Contributions

UW, HS, AW, EH, TK, and EG-S designed research. EG-S, AO-R, A-MS, LG, and MF performed research. VG, JH, RK, and PM contributed new analytic tools. EG-S and MK analyzed data. EG-S, MK, and UW wrote the paper. All authors contributed to the article and approved the submitted version.

## Funding

The study received funding from the Austrian Federal Ministry of Education, Science, and Research within the research framework in relation to the coronavirus disease 2019 pandemic (GZ 2020 0225 104). AO-R, LG, and HS acknowledge funding by the Austrian Science Fund (FWF; P 34253-B).

## Conflict of Interest

TK and MF were employed by Baxter AG. The remaining authors declare that the research was conducted in the absence of any commercial or financial relationships that could be construed as a potential conflict of interest.

## Publisher's Note

All claims expressed in this article are solely those of the authors and do not necessarily represent those of their affiliated organizations, or those of the publisher, the editors and the reviewers. Any product that may be evaluated in this article, or claim that may be made by its manufacturer, is not guaranteed or endorsed by the publisher.

## Supplementary Material

Supplementary Figure 1 Decline kinetics for NCP-specific IgG ratios (geometric mean & 95% CI) post-infection and 3 months, 6 months and 1 year thereafter; dashed gray line is negative cut-off value (ratio 0.8).

Supplementary Figure 2 **(A,B)** Correlation of log transformed decline rates of **(A)** NT_50 titers (post-infection to 1 year) and **(B)** S1-specific IgG (after completed mRNA vaccination to 5–6 months) with BMI of the investigated subjects.
