# Supplementary figures and images for: SARS-CoV-2-Specific Antibody (Ab) Levels and the Kinetic of Ab Decline Determine Ab Persistence Over 1 Year
Source: Front Med (Lausanne). 2022 Feb 15;9:822316. doi: 10.3389/fmed.2022.822316 (PMC8885586; doi:10.3389/fmed.2022.822316)

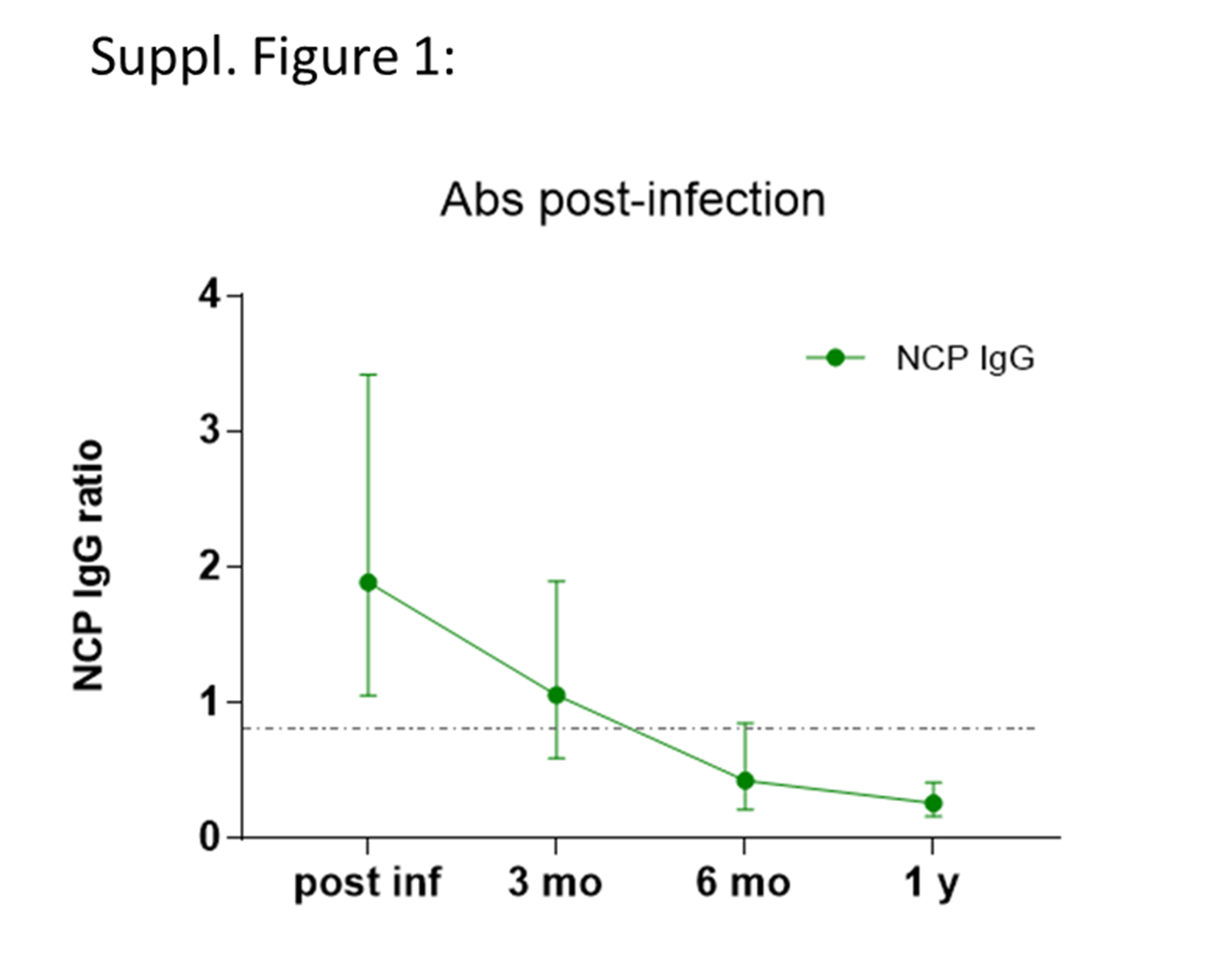

Supplement: Supplementary Figure 1 — Decline kinetics for NCP-specific IgG ratios (geometric mean & 95% CI) post-infection and 3 months, 6 months and 1 year thereafter; dashed gray line is negative cut-off value (ratio 0.8). [file Image_1.TIF]

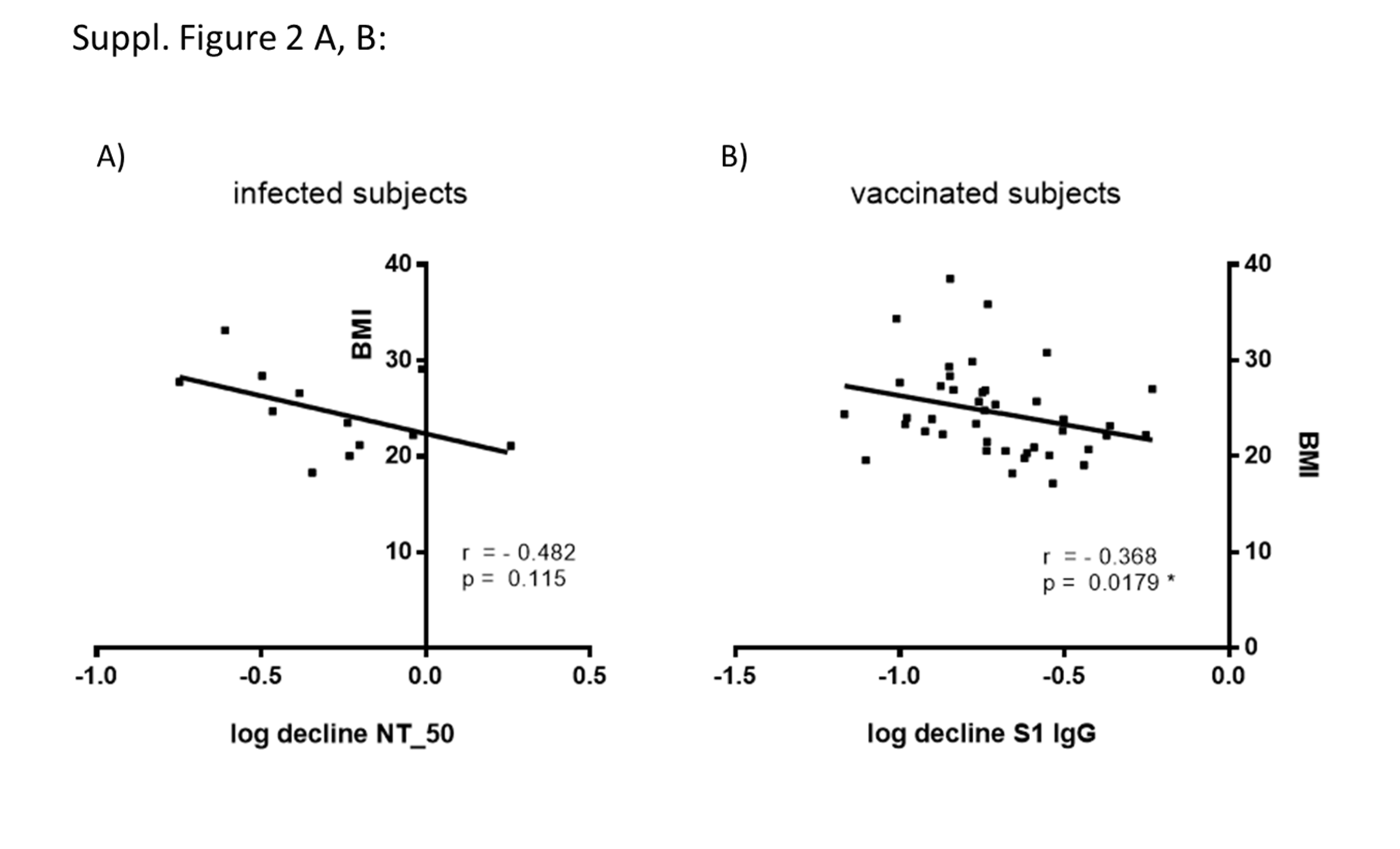

Supplement: Supplementary Figure 2 — (A,B) Correlation of log transformed decline rates of (A) NT_50 titers (post-infection to 1 year) and (B) S1-specific IgG (after completed mRNA vaccination to 5–6 months) with BMI of the investigated subjects. [file Image_2.TIF]
